# Clustering analysis of movement kinematics in reinforcement learning

**DOI:** 10.1152/jn.00229.2021

**Published:** 2021-12-22

**Authors:** Ananda Sidarta, John Komar, David J. Ostry

**Affiliations:** ^1^Rehabilitation Research Institute of Singapore, Nanyang Technological University, Singapore; ^2^National Institute of Education, Nanyang Technological University, Singapore; ^3^Department of Psychology, McGill University, Montreal, Quebec, Canada; ^4^Haskins Laboratories, New Haven, Connecticut

**Keywords:** clustering, exploration, human motor learning

## Abstract

Reinforcement learning has been used as an experimental model of motor skill acquisition, where at times movements are successful and thus reinforced. One fundamental problem is to understand how humans select exploration over exploitation during learning. The decision could be influenced by factors such as task demands and reward availability. In this study, we applied a clustering algorithm to examine how a change in the accuracy requirements of a task affected the choice of exploration over exploitation. Participants made reaching movements to an unseen target using a planar robot arm and received reward after each successful movement. For one group of participants, the width of the hidden target decreased after every other training block. For a second group, it remained constant. The clustering algorithm was applied to the kinematic data to characterize motor learning on a trial-to-trial basis as a sequence of movements, each belonging to one of the identified clusters. By the end of learning, movement trajectories across all participants converged primarily to a single cluster with the greatest number of successful trials. Within this analysis framework, we defined exploration and exploitation as types of behavior in which two successive trajectories belong to different or similar clusters, respectively. The frequency of each mode of behavior was evaluated over the course of learning. It was found that by reducing the target width, participants used a greater variety of different clusters and displayed more exploration than exploitation. Excessive exploration relative to exploitation was found to be detrimental to subsequent motor learning.

**NEW & NOTEWORTHY** The choice of exploration versus exploitation is a fundamental problem in learning new motor skills through reinforcement. In this study, we employed a data-driven approach to characterize movements on a trial-by-trial basis with an unsupervised clustering algorithm. Using this technique, we found that changes in task demands and, in particular, in the required accuracy of movements, influenced the ratio of exploration to exploitation. This analysis framework provides an attractive tool to investigate mechanisms of explorative and exploitative behavior while studying motor learning.

## Graphical abstract

**Figure:**
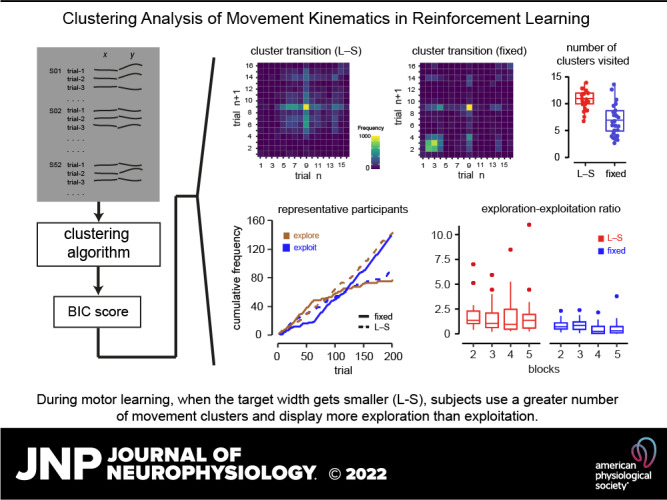

## INTRODUCTION

When learning a new motor skill, a learner often performs a series of trial and error movements to gain knowledge of the task and for action selection (1, 2). Motor skill acquisition has been modeled using reinforcement learning, where at times the movements will be successful and thus rewarded (e.g., see Refs. 3, 4, and 5). Thereafter, one has to exploit and repeat the same successful movement or vary subsequent movements to achieve better outcomes, a mode of behavior called exploration. Exploratory behavior provides an opportunity for the learner to “search” or actively gather information about the task environment (6, 7) such that appropriate actions can be developed and repeated. From a reinforcement learning perspective, motor learning can be seen as solving an exploration-exploitation dilemma, that is, to decide whether to explore or exploit in a given task environment (8). Indeed, reinforcement learning is often regarded as a decision-making sequence to determine exploration or exploitation in every action (9).

In the current study, differences in exploration and exploitation during motor learning were examined using an unsupervised machine-learning technique called clustering analysis. With this technique, movement trajectories were treated as high-dimensional data and grouped based upon similarity in movement kinematics, namely, spatial and temporal features. In this approach, exploration is defined as situations in which two consecutive movement trajectories belong to different groups or clusters (10). Conversely, exploitation is defined as repeating similar movement trajectories successively. Accordingly, the frequency of exploration and exploitation modes in a particular learning session can be computed to produce a performance measure called exploration-exploitation ratio (EER), specifically, the total number of explorations divided by the number of exploitations (10). A high value of the exploration-exploitation ratio refers to a situation in which the participant exhibited many explorations during learning, whereas a ratio equal to 1.0 indicates a balance between exploration and exploitation. This measure may shed light onto how the exploration-exploitation dilemma is resolved over time during motor learning.

We employed this unsupervised data-driven approach to address three issues. First, we aimed to characterize exploration and exploitation, and how one type of behavior was favored over the other. To examine the effectiveness of the proposed clustering algorithm, the clustering analysis was initially evaluated using simulated data with a known distribution before proceeding with the experimental (empirical) data. Second, using the experimental data set, we tested the effects of a Fixed versus a large-to-small target (L-S) condition on the choice of exploration and exploitation during training. An L-S condition was created by narrowing the size of the target zone within which the movement was considered successful. The experimental task involved arm reaching movements toward an occluded target whose size and location were unknown. Binary reward feedback was provided during training whenever the movement was successful. The reduction in target size was expected to result in a change in motor behavior as reaching movements that were originally successful might not be rewarded any longer. As a consequence, participants in the L-S condition would presumably search or perform exploration to refine their movement accuracy. Accordingly, it was hypothesized that practicing in the L-S condition would lead to more exploration than exploitation, as compared with practicing with a fixed-size target zone. Finally, on the assumption that the variability and exploration benefit motor learning in reinforcement learning contexts, we checked the idea that high exploration early in learning was related to better learning performance later on.

## MATERIALS AND METHODS

### Simulated Data Set

#### Clustering algorithm.

Clustering techniques are useful in segregating a large data set into *K* different groups or clusters without having any a priori labels. Observations within the same cluster carry the highest similarity and are sufficiently distinct from observations belonging to other groups. In the case of a high-dimensional data set, a model-based clustering technique has been shown to be robust as it takes into account the probability of each observation belonging to one of the groups or clusters (11, 12). In this technique, the data set is assumed to be generated from a mixture of *K* Gaussian distributions that are parameterized by a mean and covariance matrix. These model parameters are estimated through an iterative expectation-maximization (EM) procedure to maximize the likelihood of each observation belonging to one of the Gaussian distributions. In this manner, each distribution represents a group or cluster, and the label of each observation is subsequently assigned probabilistically to one of the clusters, such that similarity of observations within a cluster is maximized (or the variation is minimized).

In the current study, an efficient model-based clustering technique called the Fisher-EM clustering algorithm was used to classify observations (13). In each iteration, the algorithm models the data by projecting them into a new subspace such that the emerging clusters maximize the Fisher information. The output of the algorithm was the data set labeled into *K*-number of clusters. The value of *K* was then selected based on the Bayesian information criterion (BIC), which is proportional to the log-likelihood function and penalizes the model for excessive parameters. In this way, a higher BIC value represents a better fit to the data, but the incremental nature will reach a plateau. All computations were performed using the FisherEM library v. 1.5.1, with code written in R v. 3.6.3.

#### Generating simulation data.

**Figure 1.**
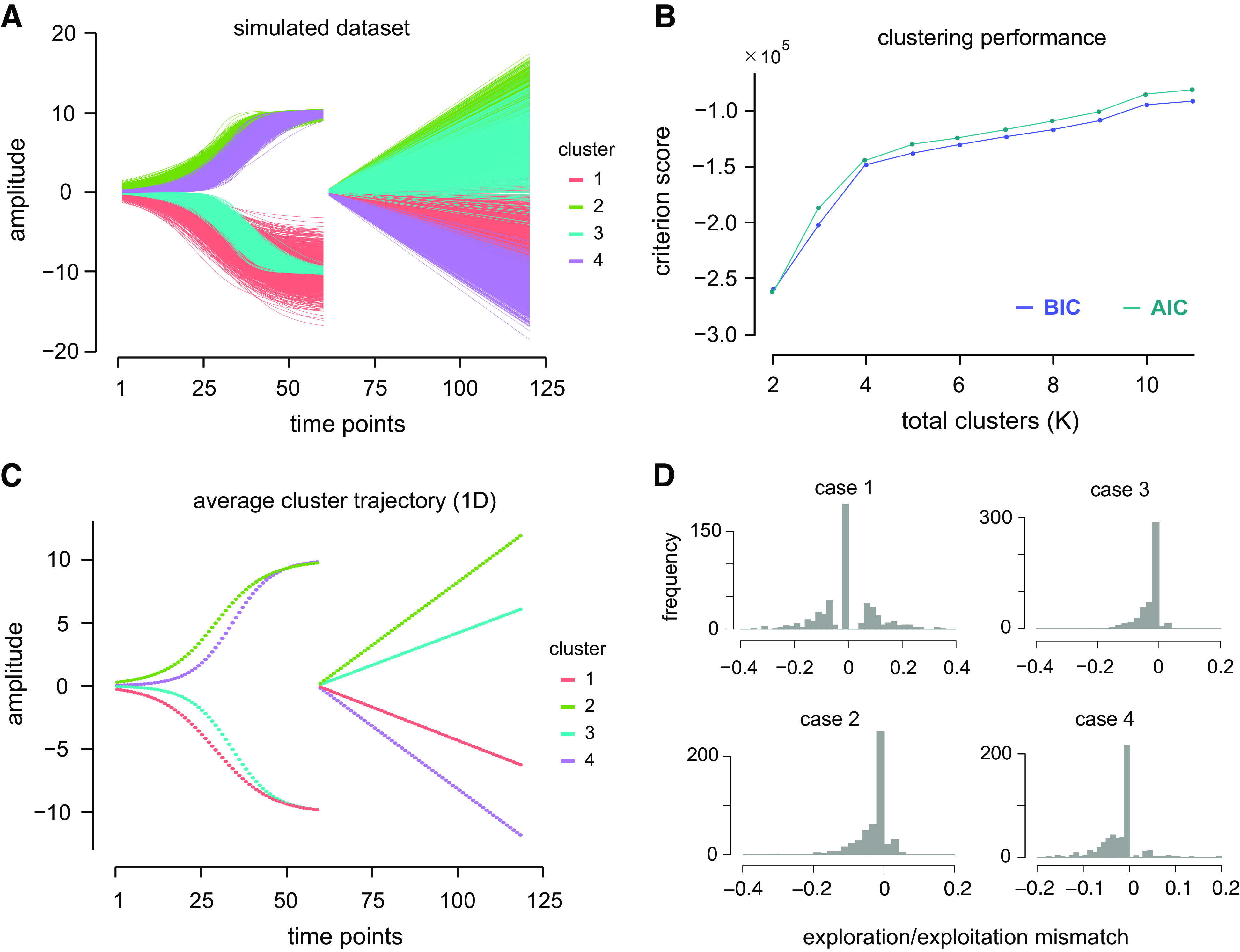
*A*: simulated data generated from a known set of distributions for the purpose of cluster validation and to test the effectiveness of the algorithm. Altogether, the data comprised *K* = 4 groups, each having *n* = 500 time-varying observations. A single observation was produced by an exponential function that was concatenated temporally with a linear function. *B*: from the clustering procedure on the simulated data set, the optimum number of clusters was selected as the first instance at which the Bayesian information criterion (BIC) approached a plateau at *K* = 4, a value that corresponds to the original *K* in the simulated data set. As a comparison, the Akaike information criterion (AIC) was placed alongside the BIC curve showing a comparable pattern. It is seen that there is reduced gain in the values of the criterion beyond this point. *C*: mean data set of each cluster for *K* = 4. Although the end points of the two exponential curves overlapped, they can be assigned to separate clusters if they differ in shape. *D*: distribution of the difference or mismatch between the true and estimated exploration-exploitation ratio (EER) for the simulated data set after bootstrapping. The high proportion of values centered around zero demonstrates that the clustering algorithm was able to produce exploration/exploitation values that are comparable with the ground truth.

As a means to validate the clustering algorithm and to better understand its efficacy, we generated a simulated data set comprising four predefined groups, each consisting of *n* = 1,500 observations with a certain distribution. Every observation had *t* = 120 time points, generated from two functions, an exponential function and a linear curve, that were concatenated temporally as follows:

$$f_{k}\left(n,t\right)=\{\begin{array}{c} a_{k}/\left(1+\exp\;\left(-\frac{t-b_{k}}{c_{k}}\right)\right),\;\;\;\;1\leq t\leq 60\\ \;\;\;\;m_{k}t,\;\;\;\;\;\;\;\;\;\;\;\;\;61\leq t\leq 120 \end{array},$$

where *a*, *b*, and *c* are related to the shape of the exponential curves, *m* is the linear slope, and subscript *k* = 1, 2, …, *K* denotes the group label. The values of *a*, *b*, *c*, and *m* of each group were sampled from a Gaussian distribution with a certain mean and variability (Table 1). Exponential functions were used as they resembled the one-dimensional time-position data observed in the actual reaching movement. To inject some complexity, the data set was concatenated with linear curves with extensive overlap, which results in a graph as depicted in Fig. 1*A*. Different colors in the graph represent the *k* different groups from which the data were generated. The degree of overlap seen in the upper and lower quadrants was deliberate, to assess whether the algorithm was able to identify *K* = 4 emerging clusters or misclassified the number of clusters to be only *K* = 2.

**Table 1.** Means ± standard deviation of Gaussian distributions used to generate each observation in the data set

*k*	*a*	*b*	*c*	*m*
1	−10 ± 2	30 ± 3	8 ± 1	−0.1 ± 0.05
2	10 ± 0.1	30 ± 2	8 ± 2	0.2 ± 0.03
3	−10 ± 0.1	35 ± 2	6 ± 1	0.1 ± 0.05
4	10 ± 0.1	35 ± 2	6 ± 1	−0.2 ± 0.03

Parameters *a*, *b*, and *c* determine the shape and curvature of the logistic curves, whereas *m* refers to the slope of the linear functions.

#### Cluster validation with simulation.

The cluster validation procedure consisted of two parts. First, the level of agreement was assessed between the cluster assignment produced by the Fisher-EM algorithm and the known ground truth labels. From the generated data set, we randomly sampled 200 trials four times to construct four hypothetical cases of behavior. Each behavior had its own number of clusters and degree of exploration according to the sampling rules indicated in Table 2. With this, we simulated people who displayed high exploration and those who did not. At the same time, the ground truth label for each data point could be known or assigned a priori. To determine the performance of the algorithm in the simulation against the ground truth, the Adjusted Rand Index (14) was used. The index essentially looks into all pairs of observations and counts both agreement and disagreement (mismatch) between the emerging clusters and the ground truth cluster labels. A value of 1.0 indicates that the algorithm groups the data in the same manner as the ground truth, whereas a value of zero means that the grouping does not agree with the ground truth. In the second part, the effectiveness of the clustering algorithm in quantifying exploration and exploitation was assessed. Within the clustering framework, one is said to be in exploitation mode in the current trial *n* when the movement belongs to the same cluster as in the previous trial *n* – 1. Exploration mode, on the other hand, takes place when there is a change of movement clusters between the current trial *n* and trial *n* – 1. In this simulation, if the sampled observations originated from all four curves with equal probability (e.g., 2-4-1-3-2-4-3-1…), then we would expect the amount of exploration to be high (*case 1* in Table 2). In contrast, low exploration means that the observations belonged to only one of the clusters most of the time (e.g., 2-2-2-3-3-2-2-2…) (*case 3*). The ratio between the total number of exploration and exploitation trials (EER) was calculated based on the known cluster assignments, giving rise to the ground truth ratio values (Table 2). The same calculation was done after running the algorithm to obtain the estimated exploration-exploitation ratio.

**Table 2.** Characteristics of different cases of hypothetical participants showing ground truth values for the total clusters K and the exploration-exploitation ratio, as well as the degree of agreement (Adjusted Rand Index) and the estimated ratio from the clustering analysis

	Sampling Rules	*K*	Agreement	EER		
				Ground Truth	Estimated	Difference
1	Data from *f_k_* were uniformly sampled to give a high level of exploration	4	97.52%	3.06 (2.27, 4.17)	3.05 (2.21, 4.23)	0.01 (−0.23, 0.24)
2	One cluster had a 70% chance of being sampled, the remaining had 10% chance; moderate exploration	4	97.28%	0.94 (0.64, 1.28)	0.97 (0.65, 1.37)	−0.04 (−0.16, 0.02)
3	Two of the four clusters had 80% and 20% chance to be sampled; low exploration	2	96.39%	0.48 (0.32, 0.67)	0.51 (0.33, 0.74)	−0.03 (−0.13, 0.01)
4	Two of the four clusters had equal chance (50%) to be sampled; balance between explore vs. exploit	2	97.69%	0.99 (0.76, 1.31)	1.02 (0.76, 1.38)	−0.03 (−0.12, 0.05)

The index assesses the proportion of time the true clusters and the emerging clusters from the algorithm agree. All values are the average statistics obtained from bootstrapping with 500 repetitions. The range in the brackets is the 95% confidence interval. EER, exploration-exploitation ratio.

To cover all possible combinations of observations in the simulated data set, bootstrapping was done 500 times. In each bootstrap sample, the degree of similarity and the frequency of exploration and exploitation were computed, and the difference (or mismatch) between the true and estimated exploration-exploitation ratio were calculated. Finally, the mean difference in the exploration-exploitation ratio was calculated with the bootstrap 95% confidence interval (CI).

### Experimental Data Set

Data were collected previously from healthy participants who have given their written informed consent and were recruited for the L-S task condition (*n* = 22, 14 females, *M* age = 22.5 yr old, SD = 3.19) (15) and for the Fixed condition (*n* = 30, 6 males, *M* age = 22.11 yr old, SD = 2.85) (16). All participants were right-handed without prior physical or neurological conditions. The experiment used a two degree-of-freedom robotic manipulandum (Interactive Motion Technologies).

#### Behavioral tasks.

**Figure 2.**
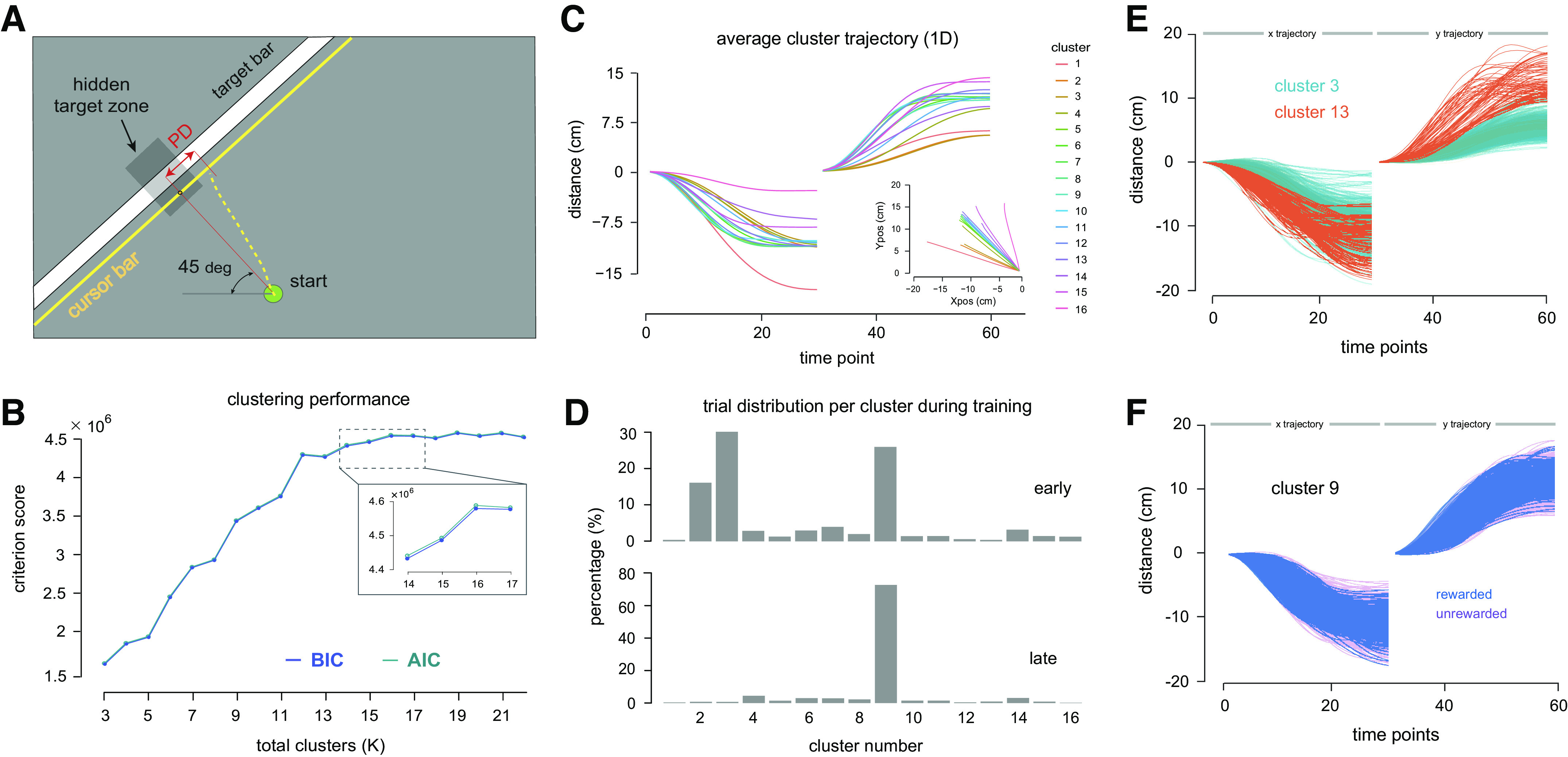
*A*: task interface of the experiment. Participants were not able to see the cursor or their arm while moving. Instead, a thin yellow line parallel to the target bar was shown. No information about the lateral position of the hand was available at any point in the session. The width of the hidden target was manipulated in the large-to-small target (L-S) condition to increase demands on movement accuracy. PD, perpendicular deviation at movement endpoint. *B*: same as Fig. 1*B*, but the input to the clustering algorithm now came from the actual behavioral experiments. The optimum number of clusters was selected at *K* = 16. Both curves suggest that there is little gain in the Bayesian information criterion (BIC) and Akaike information criterion (AIC) values beyond this point. *C*: mean trajectory of each cluster over time. Data of both axes were concatenated together as a vector, where the first 30 data points are the *X* positions and the subsequent 30 data points are the *Y* positions. It can be seen that the mean trajectory of every cluster has its own unique spatial and temporal features in each axis. The output of the clustering algorithm was movements that were grouped into different clusters according to kinematic features. In the *inset*, mean trajectory of each cluster in a two-dimensional space is shown. *D*: proportion of training trials in each cluster in *blocks 1* and *2* (*top*), and *blocks 3* and *4* (*bottom*). It can be seen that *cluster 9* comprised the greatest number of trials at the end of training (75%), up from 26% during the earlier blocks of training. Movements in this cluster also received the greatest amount of reward. In both *C* and *D*, the cluster numbering was ordered in such a way that *cluster 1* and *cluster 16* refer to movements deviated to the left and right of the target zone, respectively. *E*: trajectories belonging to two different clusters resulted from the clustering analysis. Similarity and distinction in the curvature in either *X* or *Y* axis or both contributed to the difference in the cluster assignment. The graph also depicts some degree of variation in the extent of similarity within a particular movement cluster. *F*: trajectories of *cluster 9*, showing successful (and thus, rewarded) trials superimposed on unsuccessful movements. All trajectories in the figure were aligned to the onset of movement.

Participants were seated in front of the robot with their right hand holding the robot handle at the end-effector, the shoulder abducted to ∼70° and the elbow supported by an air sled. Vision of the arm and the robot handle were blocked by a semipolarized mirror. On the display screen, a circle (20 mm in diameter) that served as a start position was presented in front of the body midline. Each participant was presented with a 1-cm thick white target stripe in the left part of the workspace, within which there was a hidden rectangular target zone (see *Target zone*). The center of the target zone was 15 cm from the center of the start circle. A thin yellow line ran parallel to the target stripe and indicated the distance of the handle from the stripe. A small 12 mm diameter yellow circle that was attached to this yellow line corresponded to the hand position. This circle was shown briefly at the beginning of each movement and disappeared as soon as the robot handle left the start position. Crucially, no error information associated with the lateral deviation of the hand from the target was provided (Fig. 2*A*). Each trial began with the presentation of a visual cue, and the movement had to terminate within the stripe and be completed within 500–700 ms. The participants were given verbal feedback about movement speed if they were consistently too slow or too fast, or if the reaching movement consistently ended outside of the stripe. Once the movement ended, the robot brought the hand back to the start position.

Participants were neither told the location of the target center nor the width of the target zone. They were instructed to reach 45° to the left and were told to learn which reaching movement was successful. Movements were considered successful if the lateral perpendicular deviation at the movement end point was within the span of the target zone; success was not based on either movement distance or speed. At the end of each successful trial, performance feedback was provided in the form of an animated explosion along with a “Nice shot!” message and a pleasant tone. The feedback appeared in an empty space within the field of view of the participant. No feedback was given for unsuccessful movements. Participants were told to complete as many successful movements as possible. In total, the participants did four training blocks of 50 trials each, separated by a short break between successive blocks. The experiment began and ended with baseline and final blocks in which no feedback was given regarding movement accuracy.

#### Target zone.

During training, movements that ended within the target zone were considered successful. The width of the target zone determined the nature of the task demands in this study. In the L-S condition, the width changed from one training block to the next (i.e., every 50 trials), while keeping the center position fixed. Here, participants were challenged with an increasing level of required movement precision. In the first training block, the size of the hidden target zone was set to the lateral range within which 50% of each participant’s baseline movements ended (baseline SD: 0.89 cm, averaged across all participants). The width would ensure that at least half of the movements would be successful. In the second block, the size was set to half the value between the first and the last target width. The range of the target widths across participants was 1.10–13.56 cm for the first block and 0.80–7.18 cm for the second block. Eventually, a target width of 0.80 cm was used for the last two training blocks and was the same for all participants. In contrast, the target width in the Fixed condition was maintained at 1.00 cm throughout training.

#### Data preparation.

The time-varying position of the robot handle in two-dimensional space was sampled at 400 Hz. For each movement trajectory, *X* and *Y* positions were time-normalized to 30 time points and then concatenated to form a row vector of 60 elements. Time normalization into a smaller sample was done to maintain an equal number of time points for each trial, while reducing the computational load. Thereafter, a complete set of row vectors from all experimental blocks of a single participant was formed, i.e., each row representing the movement trajectory for an individual trial. The same process was repeated for all participants for both task conditions together (*n*_tot_), yielding a large matrix comprising *n*_tot_ × *m*_trials_ rows and 60 columns. Thus, each row in the matrix represents an observation that consists of 60 highly correlated features derived from the continuous movement trajectory data. The Fisher-EM algorithm was applied to this large data set from all participants, where the resulting output was movement data labeled into *K*-number of clusters. The analyses were repeated for different values of *K* and the model performance was evaluated by the criterion (BIC).

#### Statistical analyses.

The output of the clustering analysis was a set of movements that were assigned to different clusters, each representing distinct spatial and temporal features in one-dimensional space (*X* and *Y* axes). Each participant might display a different number of movement clusters while learning. For example, movements of one participant might be grouped to only *K* = 3 clusters (e.g., cluster number 2, 5, and 6), whereas another participant might use a total of *K* = 8 different clusters. The average number of clusters that the participants produced in each experimental condition was quantified. To see if the two conditions exhibited a comparable number of visited clusters over the course of learning, a nonparametric Wilcoxon rank-sum test was conducted, with effect size reported as *r*. As in the simulated data set, we also quantified the amount of exploration and exploitation. When assumptions for normality and homogeneity of variance were not met for the empirical data set, nonparametric tests were used instead. To evaluate whether different task demands (i.e., the target width) influenced exploratory behavior over the course of the four training blocks, the exploration-exploitation ratio was compared using the Aligned Rank Transform for nonparametric analysis of variance (ANOVA) (17), with training block as a within-subject factor and experimental condition (Fixed vs. L-S) as a between-subject factor. The same rank-based two-way ANOVA was employed to evaluate differences in the number of successful trials between the L-S and Fixed training conditions. Effect sizes for all ANOVA are reported as partial eta-squared value (${\text{η}}_{\text{p}}^{2}$). *Training blocks 1* and *2* are referred to as the “early” learning stage, whereas *blocks 3* and *4* are the “late” learning stage. Relevant post hoc analyses were performed with Holm–Bonferroni correction for multiple comparisons. The number of successful trials was also used to evaluate any relationship between the amount of reward and the exploration-exploitation ratio. Notably, the effect of exploration at the beginning of learning on the number of successful trials later in learning was examined. Pearson’s product-moment coefficient was reported for correlational analyses with 95% confidence intervals. Statistical differences were considered significant if *P* < 0.05.

## RESULTS

### Simulated Data Set

The goals of the simulation were to assess: *1*) whether the algorithm performed the grouping as expected, and *2*) whether the exploration-exploitation ratio was comparable with the ground truth ratio. In the graph depicted in Fig. 1*B*, it is seen that there is a progressive improvement in the criterion score with more number of clusters, with the curve approaching a plateau at *K* = 4. As the data set was generated from *K* = 4 known distributions, this suggests that the algorithm was able to capture this feature of the data. The cluster validation results are summarized in Table 2. It was found that the algorithm identified clusters that matched the ground truth >96% of the time, which was equivalent to less than 8 mismatched cluster assignments out of 200 observations. In terms of the ratio between exploration and exploitation, we found overlapping values between the ground truth and estimated ratios as shown by the distribution of difference between the two values. A high proportion of zero difference values can be seen in all four behavior scenarios indicating good correspondence between actual and estimated ratios (Fig. 1*D*). Overall, these results demonstrate that the clustering algorithm is sensitive enough to correctly separate the produced data set into groups, and to estimate explorative and exploitative behaviors, respectively.

### Experimental Data Set

Using the clustering algorithm, reaching movements of both task conditions together were assigned to different clusters. From the clustering analysis, the presence of *K* emerging clusters was observed, where *K* ∈ [3, 22] potential clusters were identified within the whole data set. The BIC criterion was used to select a value of *K* that best fitted the data. Figure 2*B* illustrates the BIC values for different numbers of emerging clusters, together with the Akaike information criterion (AIC) as a comparison. The value *K* = 16 was chosen as it corresponded to the first instance at which the BIC curve reached a plateau. The same approach was taken when dealing with the simulation data. It can be seen that there was little gain in the BIC and AIC values beyond this point. The variance of each cluster can be obtained from the variance-covariance matrix of the clustering algorithm. Table 3 shows the cluster variance for *K* = 16.

**Table 3.** Variance of each cluster for K = 16 (values × 10^−2^)

1	2	3	4	5	6	7	8	9	10	11	12	13	14	15	16
5.5	1.2	1.9	2.5	3.3	2.6	1.6	2.6	1.7	5.0	2.3	2.6	2.5	2.2	1.7	1.2

In Fig. 2*C*, the mean hand movement trajectory of each cluster is shown over normalized time in each of *X* and *Y* dimensions separately. It can be seen that each movement cluster has distinct temporal and spatial features that are reflected in terms of shapes and curvatures. The *inset* of Fig. 2*C* illustrates the mean hand path of each cluster in two-dimensional space, where all trajectories are directed toward the target stripe at the left side of the workspace. The cluster numbering is indicated in sequence, where the smaller numbers are for movement trajectories nearest to the horizontal *X* axis. One notable observation from the *inset* is that several mean movement paths look quite similar. This is indeed possible as clustering took into consideration kinematic information of the whole time-varying trajectory in the *X* and *Y* axes respectively, and not merely the end point location of the movements. In other words, two different movements that ended more or less within the vicinity of the target zone might be grouped into different clusters if they showed distinct spatial and temporal features (or information) in each axis.

The mean path of *cluster 9* to *cluster 12* had one of the smallest values in terms of the lateral perpendicular deviation at movement end point relative to the center of the target, namely, 2.60, 3.00, 3.50, and 4.30 mm, respectively. *Cluster 9* comprised the largest number of movements (50.8%), and also had the most successful trials (57.4%) over the entire course of training. The distribution of trials belonging to each cluster within the first and last two blocks of training is shown in Fig. 2*D*. It was observed that some movements deviated toward the left of the target zone during the initial part of learning, which contributed to a high proportion of trials in *clusters 2* and *3*. The frequency of these movements decreased with training and in the last two blocks 75% of the trials belonged to *cluster 9*, consistent with the idea that participants produced more successful movements over the course of training. To illustrate the similarity within the same cluster and differences between two separate clusters, trajectories belonging to two exemplary clusters together with the average trajectory are reported in Fig. 2*E*. At the end of learning, all participants performed reaching movements with trajectories that were grouped into one stable cluster (*cluster 9*). Successful trajectories of this cluster are depicted in Fig. 2*F*, superimposed on trajectories that are unsuccessful. It can be seen that even in this cluster there are both successful and unsuccessful trials. Participants might perform reaching movements that were statistically similar but failed to be within the rewarded target zone and thus deemed unsuccessful.

**Figure 3.**
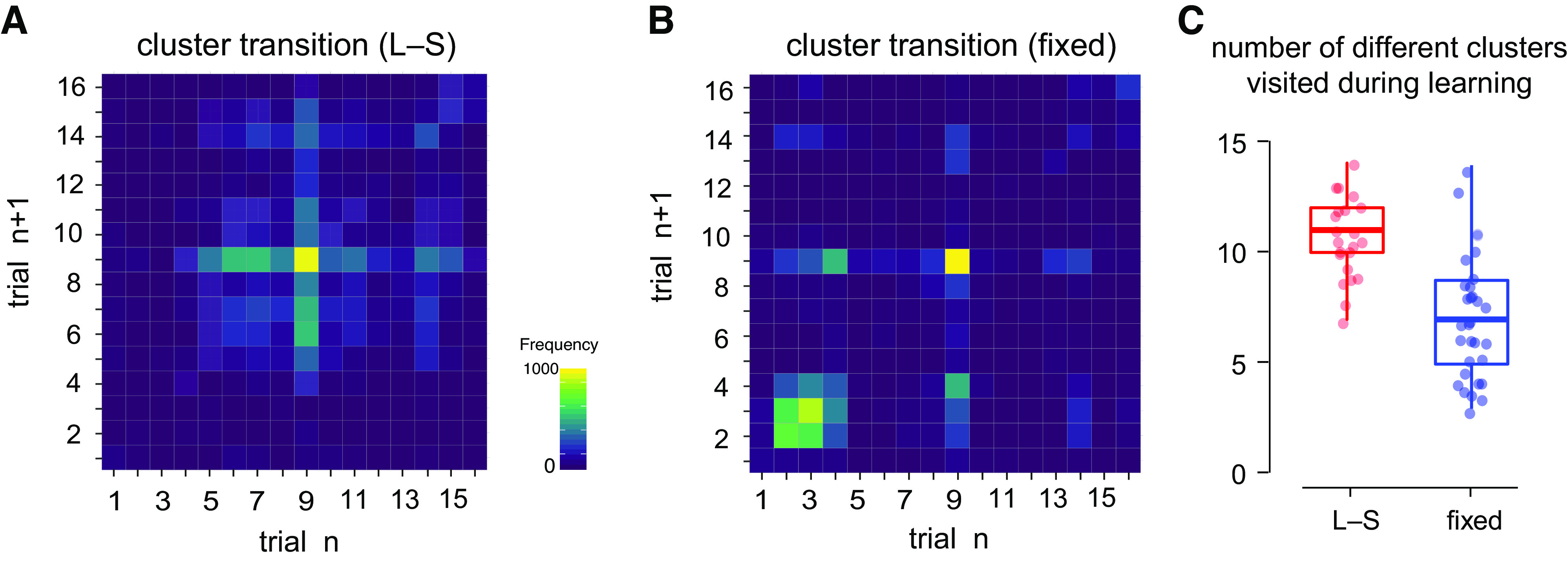
*A* and *B*: cluster transition diagram of the large-to-small target (L-S) and Fixed conditions over all training blocks. Each square represents the frequency of transition from one cluster of trial *n* (*X* axis) to another cluster in the subsequent trial *n* + 1 (*Y* axis). *Cluster 9* corresponds to the set movements that carried the greatest number of successful trials. The legend is in a logarithmic scale. *C*: overall number of clusters used over the course of training. Data from the L-S and Fixed conditions are presented in red and blue. Participants in the L-S condition had more varied movements than those in the Fixed condition, likely due to the different target widths.

We examined how reaching movement progressed on a trial-by-trial basis. The transition between clusters from one trial to the next can be visualized using a cluster transition diagram, which displays the cluster transition frequency from trial *n* to trial *n* + 1, as shown in Fig. 3, *A* and *B*. The color of each box represents the total number of times a particular transition took place in each experimental condition. It can be seen that participants in the L-S condition showed movement transitions to a greater diversity of clusters than participants in the Fixed condition, as indicated by the lighter color overall in Fig. 3*A*. In contrast, the majority of movement transitions in the Fixed condition (Fig. 3*B*) were limited to those between *clusters 2* to *4* and *cluster 9*. The high frequency of transitions between *cluster 2* and *cluster 3* were driven primarily by participants who were initially unable to find the occluded target zone. These erroneous movements also contributed to the high proportion of trials in both clusters at the beginning of learning (Fig. 2*D*).

While learning, individual participants might produce movements that fall into differing numbers of clusters. We examined for each participant the number of movement clusters visited at least once, and whether this observation was influenced by the difference in the experimental conditions. Out of 16 emerging clusters, participants in the L-S condition on average visited a greater number of clusters than those in the Fixed condition (Wilcoxon rank-sum test with continuity correction, *W* = 574, *P* < 0.001, *r* = 0.63). The difference is illustrated clearly in Fig. 3*C*. As each cluster carries certain kinematic features, this finding suggests that narrowing target width is accompanied by a greater diversity of movement kinematics.

The width of the target zone in the L-S condition decreased between *blocks 1* and *2* and again between *blocks 2* and *3*, which was equivalent to requiring increased task precision. In the Fixed condition, on the other hand, the same target width was maintained throughout training. Although, early in learning, L-S training resulted in more rewarded trials than Fixed training (presumably as a result of the larger initial target width), the latter was found to achieve more rewarded trials late in learning [block × condition interaction, *F*(3,150) = 31.34, *P* < 0.001, ${\text{η}}_{\text{p}}^{2}$ = 0.38]. The average number of rewarded trials (out of 200 trials) across the four training blocks was 31.86, 28.13, 10.70, and 14.00 for the L-S condition, and 14.17, 14.80, 17.70, and 17.4 for the Fixed condition. In terms of total rewarded trials between both conditions, the L-S condition had higher number than the Fixed condition [*F*(1,50) = 5.51, *P* = 0.023, ${\text{η}}_{\text{p}}^{2}$ = 0.09]. All participants with more reward in the first two training blocks also attained more successful trials late in learning, although this relationship is weaker for the L-S condition [Fixed: *r* = 0.61, *P* < 0.001, confidence interval (CI: 0.32, 0.80); L-S: *r* = 0.44, *P* = 0.04, CI (0.03, 0.72)].

**Figure 4.**
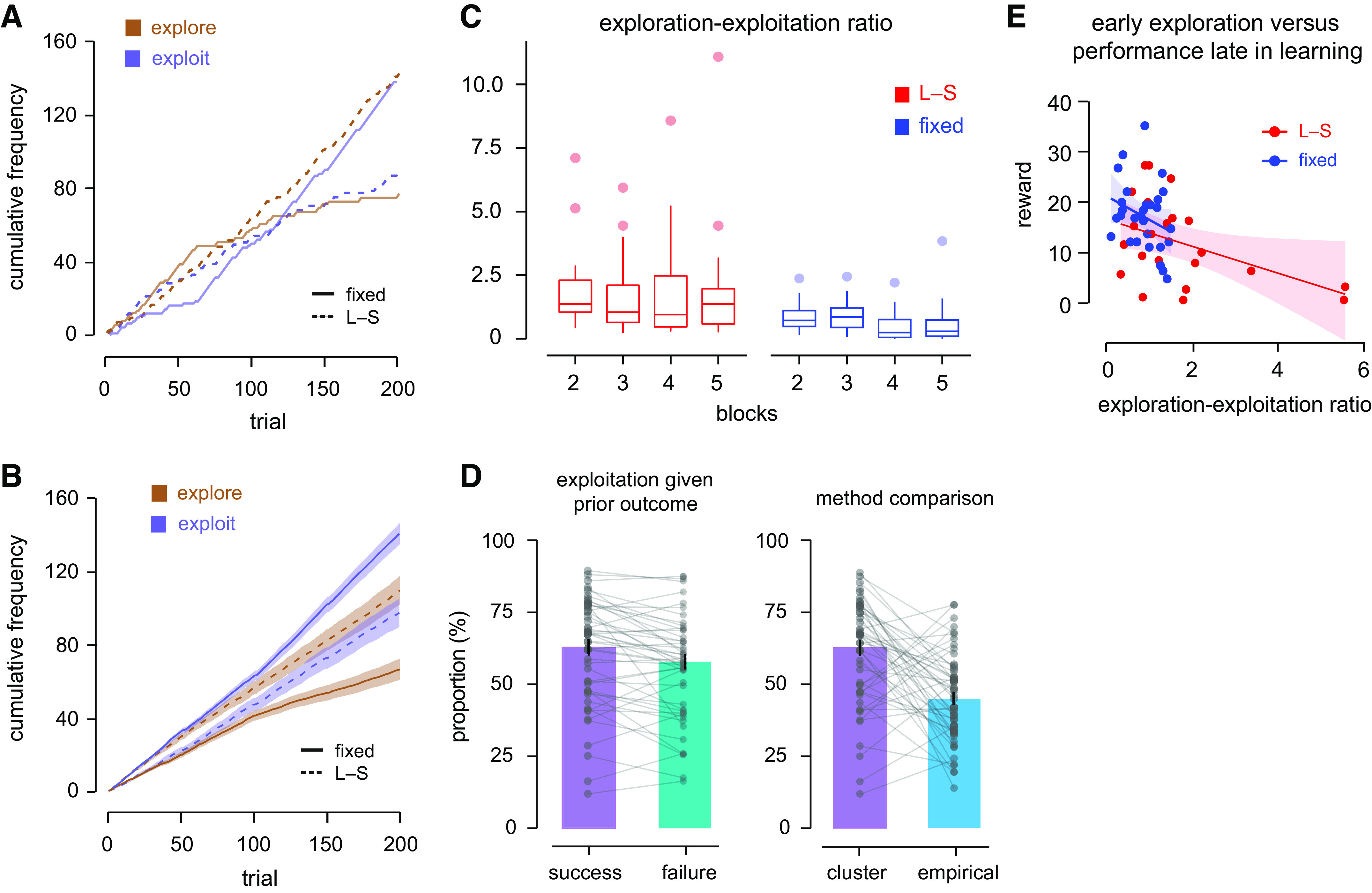
*A*: cumulative frequency of exploration and exploitation of a representative participant from each experimental condition. The diagram shows the progression of each behavior over time during motor learning, and how one mode of behavior occurs more frequently than the other. *B*: same as *A*, but the cumulative frequency was averaged across all participants in each experimental condition. The shaded color represents the standard error. *C*: to characterize differences between experimental conditions in the choice of exploration over exploitation, a performance measure called exploration-exploitation ratio (EER) was computed early and late in learning (first two vs. last two blocks). On average, participants in the L-S condition used more exploration than exploitation as compared with those in the Fixed condition (see main text). *D*: repeating movements from the same cluster is termed exploitation. In the *left* panel, the proportion of trials in which there is exploitation following a rewarded trial is reliably greater than the proportion of trials in which exploitation occurs following failure. In the *right* panel, the proportion of trials in which there is exploitation following a rewarded trial is compared with the empirical calculation, which is defined as the proportion of trials in which a rewarded movement is repeated and rewarded for the second time. *E*: more exploration early in learning (first two blocks) was accompanied by fewer rewarded trials late in learning (last two blocks) in the large-to-small target (L-S) condition.

Figure 4*A* depicts the cumulative frequency of exploration and exploitation over time for a representative participant from the Fixed (*top*) and L-S (*bottom*) conditions. The participant in the Fixed condition initially produced more movements that were classified as exploration than exploitation, but this choice of behavior flipped by the end of learning, presumably because of a better ability to locate the hidden target successfully. This is in contrast with the L-S condition where exploration was greater than exploitation through to the end of learning. Figure 4*B* shows the cumulative frequency of exploration and exploitation averaged over all participants in each condition. As learning progressed, participants belonging to the Fixed condition showed more exploitation, whereas in the L-S condition, exploration remained greater when the target width changed.

To assess how exploration and exploitation differed in the two experimental conditions during learning, we computed for each participant the number of movements classified as exploration versus exploitation. The ratio of the number of exploration to exploitation, denoted as the exploration-exploitation ratio, was used to investigate how the two different modes of behavior evolved across four training blocks. There were reliable differences between the two experimental conditions [*F*(1,50) = 15.95, *P* < 0.001, ${\text{η}}_{\text{p}}^{2}$ = 0.24] and between four training blocks [*F*(3,150) = 3.64, *P* = 0.01, ${\text{η}}_{\text{p}}^{2}$ = 0.07]. The variation of exploration-exploitation ratio across training blocks is different between the two conditions [block × condition interaction, *F*(3,150) = 2.83, *P* = 0.04, ${\text{η}}_{\text{p}}^{2}$ = 0.05]. Specifically, although the Fixed condition shows a reliable reduction in the ratio across blocks [Kruskal–Wallis test, *H*(3) = 19.21, *P* < 0.001, η^2^ = 0.14], the exploration-exploitation ratio in the L-S condition remained high through to the end of training [*H*(3) = 1.57, *P* = 0.67, η^2^ = 0.01] (Fig. 4*C*). The narrowing of target width presumably required the participants to maintain a high level of exploration. In contrast, the lower exploration-exploitation ratio values in the Fixed condition are consistent with the fact that the amount of exploitation exceeded exploration (Fig. 4*B*, Fixed task).

Participants in the L-S task who had higher exploration-exploitation ratio values in the first two blocks of training continued to have higher exploration-exploitation ratio in the last blocks [*r* = 0.80, *P* < 0.001, CI (0.69, 0.88)]. Taking all participants together, those who had a greater number of movement clusters during training also displayed higher exploration-exploitation ratios [*r* = 0.62, *P* < 0.001, CI (0.43, 0.76)]. The ratio between exploration and exploitation for each individual may influence reward performance during training. One possibility is that participants who initially displayed more exploratory behavior might have a greater number of rewarded trials late in learning. Alternatively, excessive exploration may be maladaptive leading to worse performance overall late in learning. To test these possibilities, the average exploration-exploitation ratio early in learning (first and second training blocks) and the total rewarded trials late in learning (third and last training blocks) were examined across all participants. It was found that those who had higher exploration-exploitation ratio early in learning had fewer rewarded trials in the latter stage of learning [*r* = −0.43, *P* = 0.001, CI (−0.63, −0.18)] consistent with the idea that exploration in large quantities can have adverse effects on eventual learning.

In past studies, exploration and exploitation have been quantified using the change in reaching direction contingent upon the history of past reward. To check our results in relation to other methods of quantifying exploration or exploitation, additional analyses on our experimental data were conducted. The probability of exploitation given reward on the preceding trial versus exploitation given failure were found to be 62.8% and 57.8%, respectively (Fig. 4*D*, *left*). The probability of exploiting given reward is significantly greater than the probability of exploiting given failure [paired *t* test, *t*(51) = 3.56, *P* < 0.001, *d* = 0.25]. The complement probabilities, namely, the probability of exploration given reward and given failure, were 37.2% and 42.2%.

In another analysis, which compared the empirical data and the clustering approach, we assessed the amount of exploitation as defined by the clustering algorithm with that observed in the empirical data following a rewarded movement (without any clustering). By definition, exploitation happens when one repeats the same action, presumably to maximize what one has gained at the moment (8). We calculated a measure that fulfills the definition empirically, i.e., the proportion of trials in which the current movement is successful or rewarded, given a rewarded movement on the preceding trial. We then computed a comparable measure for the clustering analysis, the proportion of cases in which two successive movements belong to the same cluster after the first was rewarded. The right diagram in Fig. 4*D* shows that exploitation based on empirical data is significantly lower than that estimated using the clustering analysis [62.8% vs. 45.0%, paired *t* test, *t*(51) = 4.91, *P* < 0.001, *d* = 0.68]. This means that following a rewarded trial, the subsequent movement may be similar enough kinematically to fall into the same cluster but may miss the target zone in the actual experiment, and hence may not be rewarded. The clustering analysis thus suggests that the usual way of measuring exploitation, based on the repetition of successful movements, may underestimate how much repetition actually occurs.

One approach to quantify the amount of exploration and exploitation in the empirical data set is to compute the trial-to-trial change in direction (Δ*m*) as a function of the preceding reward outcome (success/failure). In this method, exploration is estimated by the average Δ*m* following an unsuccessful outcome in the preceding trial. We conducted this analysis at two different points during the movement. First, the change in direction was estimated by the trial-to-trial difference in perpendicular deviation (PD) at movement end point. We then assessed whether the amount of exploration using this approach was related to the exploration-exploitation ratio obtained by the clustering method, but no reliable relationship was observed [*r* = 0.13, *P* = 0.34, CI (−0.142, 0.392)]. In a second analysis, the change in direction Δ*m* was estimated by the trial-to-trial difference in average perpendicular deviation measured at midflight, which was an average value taken over the middle third of the movement. It was found that participants who had a higher midflight variability also had a higher exploration-exploitation ratio [*r* = 0.53, *P* < 0.001, CI (0.29, 0.69)]. This result suggests that the measure of exploration obtained from the clustering captures patterns of movement direction at mid-flight rather than at movement end point.

In a final analysis, we quantified the average change in movement direction in terms of the magnitude of shift between clusters following successful and unsuccessful trials. This provided an ordinal measure of the magnitude of the change in movement direction since we had sorted the clusters numerically in ascending order from left to right. Thus, the larger the magnitude of the shift between clusters, the greater the change in movement direction. The aim of this analysis, like that earlier, was to estimate the relative magnitude of movement change contingent upon the preceding reward, but in the context of clustering. Our finding indicates that the average cluster shift after an unsuccessful trial was greater than the one following a rewarded trial [paired *t* test, *t*(51) = 3.81, *P* < 0.001, *d* = 0.28]. This measure resembles trial-to-trial movement variability (Δ*m*), whose magnitude was found to be dependent upon the preceding reward. To summarize, these additional analyses suggest that the clustering method is able to capture, not only kinematic characteristics of each trajectory, but also the effect of prior trial outcome on movement during learning.

To assess the robustness and consistency of the main findings for different total numbers of clusters, a sensitivity analysis was conducted in which the aforementioned analyses were repeated using the remaining values of *K*, from 3 to 22. It was found that participants in the Fixed group performed significantly smaller number of clusters with a large effect size for all *K* > 7. A reliable difference in exploration-exploitation ratio between the Fixed and L-S conditions was found for all *K* values, with a medium to large effect size. Compared with earlier blocks, participants on average explored less late in learning, but statistically significant differences are seen only in some values of *K* between 13 and 18. An interaction effect with a small effect size can be seen for some values of *K*. Exploration early in learning is negatively correlated with reward performance late in learning for all *K* > 10. The full outcomes of the sensitivity analyses can be found in Supplemental Table S1; see https://doi.org/10.6084/m9.figshare.14618592.v2. Choosing a value of *K* with a smaller BIC might result in a loss of sensitivity, whereas a higher value of *K* that results in a marginal increase in BIC does not change the main findings.

## DISCUSSION

Reinforcement learning using binary feedback to indicate success or failure has been used in earlier work as an experimental model of human motor learning, in which we found neuroplasticity in sensorimotor and reward-related networks (e.g., see Refs. 5 and 15). In the reinforcement learning literature, it has been proposed that the choice of exploration or exploitation is dependent on the availability of reward, such that one repeats the same action to maximize the reward available (i.e., a greedy policy) (8). In behavioral studies, efforts have been made to understand and characterize the choice of exploration versus exploitation. Typically, motor variability is seen as a reflection of exploration and is thought to be essential for motor learning (18, 19). In one study, exploration was quantified as an increase in trial-to-trial changes in deviation measured at movement end following consecutive failures (15). In another study, it was found that exploration could remain elevated to facilitate learning in subsequent training blocks (20). Higher amount of exploration also occurs in conjunction with an additional task dimension or spatial complexity (21). Such changes in motor behavior may well involve explicit awareness to correct for wrong movements based on feedback (22). Accounting for motor noise when quantifying exploration from behavioral experiments is required to correctly model the behavior (23). However, the relationship between reward feedback and the associated mode of behavior is uncertain. For example, movements that look like exploration may arise either to gain better outcomes or due to the inherent variability in the sensorimotor system. Likewise, exploration may not always follow an unsuccessful outcome as the change in direction may be small. Although some recent work identified reward-related neural signatures from a person during learning itself (e.g., see Ref. 24), directly capturing the actual intentions to explore (or exploit) in real-time remains a challenge.

### Cluster-Based Approach

Clustering algorithms are unsupervised machine learning techniques that are commonly used to classify unknown patterns in the data without a priori assumptions or labels. These techniques have been adopted in previous studies of motor control and biomechanics to understand movement patterns and multijoint coordination (10, 25–28). The term “pattern” is used in machine learning to mean unique features hidden in the data that are revealed through statistical methods. In the present study, movements were grouped probabilistically based on discriminating features in movement patterns over time. The word “movement pattern” means reaching movements with different spatial and temporal features. The algorithm considered not only the end point location, but the underlying characteristics of the whole time-course in the *X* and *Y* axis. It was found that by the end of learning, movement trajectories across all participants converged primarily to a single cluster (*cluster 9*). This cluster was also the movement cluster with one of the lowest average perpendicular deviation (or error) and thus associated with the greatest number of successful trials.

The clustering algorithm separates movements not only based on the direction and end location, but also how each reaching movement was produced over the course of the trial. Consequently, spatial and temporal variations result in different curvatures or shapes, which eventually contribute to discriminating attributes during clustering. Empirically, such variations are in line with previous theories by Schmidt (as a qualitative change in motor program) (29) or Kelso (as a qualitative change in attractor) (30). Indeed, some variations can still be found between two movements despite being labeled as similar. What is more essential is how similarities and differences are defined. In the case of the model-based clustering method, it is a statistical difference between two time-varying data sets.

Broadly speaking, the clustering techniques can be implemented separately using participant-level data or using the whole data set at once as a group. What distinguishes the two approaches is the definition of similarities and differences. By grouping the individual data set separately, the basis of similarities and differences will be restricted to those from each participant and thus prevent a quantitative comparison across participants or between experimental conditions. We chose to use the latter approach of using the whole data set at once because it allows us to look at the variation between experimental conditions in addition to the individual dynamics. As a result, variation among movement clusters reflects differences in the curvature or shape of the trajectory across all participants. In the same way, trajectories that carry identical curvature across all participants were grouped together as the same cluster. This is despite the fact that some degree of variability in the extent of this similarity is present (Fig. 2, *E* and *F*). Combining all participants into one data set also allowed us to identify a stable cluster at the end of learning that is common to all participants (10).

### Exploration and Exploitation

How the movement kinematics changed on a trial-to-trial basis during training can be examined and visualized using a cluster transition diagram. Not only does the diagram tell us the frequency of transitions from one specific cluster to another, it also shows the diversity of movement clusters adopted by the participants. The clustering approach has the advantage of probing how exploration is chosen over exploitation over time by computing the ratio between the frequency of exploration and exploitation (EER) within a single session. Cluster transition may be related to the exploration-exploitation ratio in a number of ways. In principle, exploration may occur either by displaying more variety in movement kinematics (i.e., greater number of visited clusters), or by changing between one existing cluster to another (i.e., higher frequency of transition). While learning, a participant could display a lower exploration-exploitation ratio but a greater number of different clusters. For example, the movements may belong to “1-1-1-2-2-2-2-3-3-3-3-4-4-4-5-5-5-5-6-6-6-7-7-7….” Another participant might have a higher exploration-exploitation ratio, but perform a smaller variety of movement clusters, e.g., “1-2-3-2-1-3-2-3-1-2-3-2-1-2-3-2-1-2-3-1-2…,” and so on. We found that there was a reliable relationship between exploration-exploitation ratio and the overall number of clusters visited, such that people that had a greater diversity of movement clusters, also had more exploration. Although the result is correlational, this is consistent with the idea that more clusters visited corresponds to greater exploration.

Distinguishing active exploration from natural variability on the basis of behavioral data is challenging, if not impossible, particularly when movement variability and exploration overlap. In the context of the clustering analysis, we define active exploration as those cases in which the cluster switches from one trial to the next. It is therefore assumed that active exploration can be characterized probabilistically as between-cluster variation which is reflected in the spatial and temporal (kinematic) features of movements. In contrast, variation within the same cluster reflects at least in part the natural movement variability for a given motor task. However, even within a movement cluster some of this variation may be quite intentional as a form of minor exploration, which is in line with the recent work by Pacheco et al. (7). Such variation within a cluster of an individual could be associated with minor adjustment in parameters within the same motor program (e.g., see Ref. 29), for instance, in the face of a change in task environment.

### Change in Task Demand

The nature of the task in this study was determined by the target width. This approach has been followed in previous reward-based motor learning studies that affected learning performance. The task manipulation has been carried out in a number of ways. Notably, the target within which the movement would be rewarded was changed either in size or shifted in the position (15, 20, 22, 31–33). In a work by Cashaback et al., for example, different reaching directions carried different reward probability and the probability profile across direction could be either steep or more gradual. Other studies introduced a shift in the target location or pointing angle, either gradually over time (e.g., in the work by Uehara et al.) or in a more discrete fashion after a certain number of trials (e.g., in the work by Pekny et al.). In the current study, the target zone for one of the conditions became narrower between *blocks 1* and *2* and also between *blocks 2* and *3*, keeping the target center stationary. The wider initial target width required lower precision and enabled participants to produce a higher number of successful trials. This manipulation is similar to that in an errorless motor task where participants learned golf putting with an increasing distance from the hole, serving to increase the task demand (34). In contrast, participants in the Fixed condition encountered a small target width from the onset and produced fewer successful trials.

By providing larger initial target widths, participants not only produced more rewarded trials early in training, but also demonstrated more exploration than exploitation, and tended to use a greater number of movement clusters. This is contrary to the general understanding that positive feedback and reward may promote less exploration than exploitation. This seemingly contradictory finding might be explained by considering the definition of exploration and the number of successful trials. In the initial stages of learning, participants sampled many possible reaching directions with higher spatial and temporal variability. However, it has been suggested that excessive movement variability to explore different solutions in space can be unfavorable to motor learning as it adversely affects the ability to retain useful solutions (35). The adverse effects on eventual learning performance of large initial variability were likely compounded by a reduction in the width of the target zone, which increased task demands, in particular, when the variability (SD = 0.89 cm) was larger than the size of the narrowest target width (0.80 cm) in the L-S group. This may help explain why participants who performed more “switching” initially received less reward at the end. Such a situation provides an example where higher exploration relative to exploitation becomes less effective when it is accompanied by more or less the same rewarded outcomes. This seemingly maladaptive situation in turn impacted subsequent learning stages where the requirement of movement precision and accuracy increased. It is noteworthy that exploration involves gathering of reliable information about the movements which are, not only successful, but also unsuccessful (6). The findings raise a question of whether there exists an optimum level of difficulty (or in our case the target size) that allows faster motor skill learning through “safe” exploration. However, it is likely that a satisfactory difficulty level may be closely related to individual differences in learning ability and may have to be continuously adjusted during learning.

Why does the reach direction change with decreasing target width between blocks? One possibility is that the switch in direction appears as a form of exploration in search of the successful outcomes previously obtained in the preceding block. Another possibility is that changes in the motor plan gave rise to distinct clusters due to adaptation to changes in task demands, which in this case is the narrowing of the target zone. According to a prevailing theory of motor adaptation (36), adaptation occurs largely due to sensory prediction error, in which there is an anticipated or predicted sensory outcome of a movement and a systematic error is introduced into the perceived movement by, for instance, rotation of visual feedback or a mechanical perturbation to an otherwise straight movement. The distinction between error-based adaptation and reinforcement-based learning has advanced, particularly in light of recent studies that showed reinforcement-based mechanisms can occur simultaneously with and support motor adaptation (3, 4, 37–39). In the context of our task, we believe that motor learning in response to target size constriction is primarily driven by reinforcement-based processes for two reasons. First, participants did not know the exact location of the target, and thus, no directional information regarding error is available. More critically, no information regarding target width was conveyed to the participants (beyond that related to their success or failure). This imposes a challenge for adaptation-based learning because there is no clear sensory target relative to which error has to be minimized. Indeed, one study observed that when the sensory error is uncertain or unreliable, participants depend more on another learning mechanism that is based on success/failure (4). Second, because the only learning signal appeared as success/failure, participants had to reinitiate the search process to be successful and obtain a reward. This likely happens through trial and error or exploration, which is a hallmark of reinforcement learning (8).

### Limitations and Conclusion

This study has some limitations which should be noted. The width of the target zone, which was the main experimental manipulation in this study, was changed in a single direction. To fully understand the effect of varying task demands on motor learning, future work that includes an additional condition in which the target size increases is warranted. For a better comparison, the smallest width for the narrowing and widening conditions might be the same as the target width of the fixed group (1.00 cm). Another limitation is the definition of exploration/exploitation that does not depend on the history of prior reward. The unsupervised clustering approach was employed to understand the reaching movements using information from the whole trajectory. As such, exploration and exploitation may somewhat be different from the traditional view that is dependent very much on the retrospective rewarded outcomes. Still, our analyses have shown that this method is sensitive enough to differentiate between rewarded and unrewarded movements, and to understand movements that will yield the highest reward, or that are repeated most often at the end of training.

In summary, we presented an application of machine learning techniques to harness the wealth of kinematic data in motor learning studies and complement traditional statistical analyses. Using an unsupervised clustering algorithm, trajectory data were classified into different clusters and trial-to-trial transitions were used to denote exploration and exploitation. The frequency of each mode of behavior over the learning period was used to estimate how a learner chose exploration over exploitation. The choice of exploration over exploitation was found to be influenced by a change in the task demand, but excessive exploration over exploitation could be detrimental to motor learning. Motor exploration is deemed useful for learning but only when different movement leads to different trial outcomes. Overall, this data-driven analysis provides an attractive method to study mechanisms of explorative and exploitative behavior during motor learning.

## SUPPLEMENTAL DATA

10.6084/m9.figshare.14618592.v2 Supplemental Table S1: https://doi.org/10.6084/m9.figshare.14618592.v2.

## GRANTS

This work is supported by the Rehabilitation Research Institute of Singapore, Research Fellowship Program (RFP/19002).

## DISCLOSURES

No conflicts of interest, financial or otherwise, are declared by the authors.

## AUTHOR CONTRIBUTIONS

A.S. and D.J.O. conceived and designed research; A.S. performed experiments; A.S. analyzed data; A.S., J.K., and D.J.O. interpreted results of experiments; A.S. prepared figures; A.S., J.K., and D.J.O. drafted manuscript; A.S., J.K., and D.J.O. edited and revised manuscript; A.S., J.K., and D.J.O. approved final version of manuscript.
